# Stimuli-Responsive Gels

**DOI:** 10.3390/gels4030060

**Published:** 2018-07-09

**Authors:** Dirk Kuckling

**Affiliations:** Organic and Macromolecular Chemistry, Department of Chemistry, Paderborn University, Warburger Str. 100, D-33098 Paderborn, Germany; dirk.kuckling@uni-paderborn.de

Although the technological and scientific importance of functional polymers have been well established over the last few decades, the most recent focus that has attracted much attention concerns stimuli-responsive polymer gels. These materials are of particular interest due to their abilities to respond to internal and/or external chemo-physical stimuli; such responses are often large and macroscopic. Aside from the scientific challenges of designing stimuli-responsive polymer gels, the main technological interests concern numerous applications, ranging from catalysis in microsystem technology and chemo-mechanical actuators to sensors. Since the phase transition phenomenon of hydrogels is theoretically well understood, advanced materials based on predictions can be prepared. Since the volume phase transition of hydrogels is a diffusion-limited process, the size of the synthesized hydrogels is an important factor. Consistent downscaling of the gel size will result in fast smart gels with sufficient response times. To apply smart gels in microsystems and sensors, new preparation techniques for hydrogels have to be developed. For upcoming nanotechnology, nano-sized gels as actuating materials would be of great interest. Finally, new design concepts for tough polymer gels are of interest for overcoming the mechanical shortcomings of conventional gels.

This Special Issue includes 17 papers covering a wide range of subjects including thermo- and pH-responsive hydrogels, functionalized materials, supramolecular stimuli-responsive structures, composite hydrogels, sensors, and biomedical applications. Together, these contributions not only provide an excellent overview of the current state-of-the-art in the field, but also point out exciting challenges and opportunities for future work. In a number of reviews, the most recent findings on “Stimuli-Responsive Gels” are compiled in this book, also supplemented by original contributions.

Thermo-responsive gelling materials constructed from natural and synthetic polymers can be used to provide triggered action and therefore are employed in customized products such as drug delivery and regenerative medicine products, as well as in other industries. Some materials enable Arrhenius-type viscosity changes based on coil to globule transitions. Others produce more counterintuitive responses to temperature change because of agglomeration induced by enthalpic or entropic drivers. The contribution of M. Joan Taylor et al. reviews recent developments in these thermo-responsive gels (doi:10.3390/gels3010004). Poly(vinyl caprolactam) (PNVCL) is one of the most important thermo-responsive polymers because it is similar to poly(*N*-isopropyl acrylamide) (PNIPAM). The use of PNVCL instead of PNIPAM is considered advantageous because of the assumed lower toxicity of PNVCL. The review by Chang-Sik Ha et al. focuses on the recent studies on PNVCL-based stimuli responsive three-dimensional hydrogels (macro, micro, and nano) for biomedical applications (doi:10.3390/gels2010006). In order to study the impact of a novel polyphenolic-based multiacrylate on the properties of *N*-isopropylacrylamide (NIPAM) hydrogels, J. Zach Hilt et al. synthesized a series of novel temperature-responsive hydrogels with varying contents of chrysin multiacrylate (ChryMA) (doi:10.3390/gels3040040). It was shown that the incorporation of ChryMA decreased the swelling ratios of the hydrogels and shifted their lower critical solution temperature (LCST) to a lower temperature, which could be attributed to the increased hydrophobicity being introduced by the ChryMA. Kuckling et al. synthesized new functional monomers based on vanillin (doi:10.3390/gels2010003). The incorporation of a dialkyl amino group compensated for the hydrophobic effect of the vanillin monomer. PNIPAM hydrogel thin films with transition temperatures in the physiological range could be obtained.

Stimuli-responsive cationic polymers and hydrogels are an interesting class of “smart” materials that respond reversibly to changes in the external pH. This reversible swelling-shrinking property brought about by changes in external pH conditions makes these materials useful in a wide range of applications such as drug delivery systems and chemical sensors, which are summarized in a review by G. Roshan Deen et al. (doi:10.3390/gels4010013). A specific class of responsive polymer-based hydrogels, which are formed through the association of oppositely charged polyion segments, is highlighted in a review by Christine M. Papadakis and Constantinos Tsitsilianis (doi:10.3390/gels3010003). The underpinning temporary three-dimensional network is composed of hydrophilic chains (either ionic or neutral) physically crosslinked by ion pair formation arising from the intermolecular polyionic complexation of oppositely charged repeating units (polyacid/polybase ionic interactions). Due to the weak nature of the involved polyions, these hydrogels respond to pH and are sensitive to the presence of salts. Adding poly(ethylene glycol) (PEG) during the preparation of hydrogels, followed by removal after polymerization, is shown by Björn T. Stokke et al. to improve the swelling dynamics of DNA hybrid hydrogels upon specific ssDNA probe recognition, which is peculiar for these hydrogels (doi:10.3390/gels1020219). Supramolecular stimuli-responsive polymer gels were constructed by Charles-André Fustin and Jean-François Gohy et al. from heterotelechelic double hydrophilic block copolymers that incorporate thermo-responsive sequences (doi:10.3390/gels1020235). The dynamic mechanical properties of this particular class of materials are studied by rheological experiments, showing that structurally reinforcing the micellar network nodes leads to the precise tuning of the viscoelastic response and yield behavior of the material. Finally, the release behavior of glucagon-like peptide-1 (GLP-1) from a biodegradable injectable polymer (IP) hydrogel was reported by Yuichi Ohya et al. (doi:10.3390/gels3040038). This hydrogel shows temperature-responsive irreversible gelation due to the covalent bond formation through a thiol-ene reaction.

A further review by Paula I. Soares et al. presents recent advances in stimuli-responsive gels with an emphasis on functional hydrogels and microgels, highlighting the high impact of stimuli-responsive hydrogels in materials science (doi:10.3390/gels4020054). A final review by Michael J. Serpe et al. is dedicated to the development and application of hydrogels and hydrogel particles for sensing small molecules, macromolecules, and biomolecules (doi:10.3390/gels2010008). For usage in microsystems, the preparation, and hence the characteristics, of these hydrogels (e.g., degree of swelling, size, cooperative diffusion coefficient) are key features, and have to be as reproducible as possible. Richter et al. focuses on the preparation reproducibility of PNIPAAm hydrogels under different conditions, and investigates the influence of oxygen and the UV light source during the photo polymerization process (doi:10.3390/gels2010010). T. Randall Lee et al. describes the encapsulation of gold nanoshells (~160 nm in diameter) within a shell of temperature-responsive poly(*N*-isopropylacrylamide-*co*-acrylic acid) (P(NIPAM-*co*-AA)) using a surface-bound rationally-designed free radical initiator in water for the development of a photo thermally-induced drug delivery system (doi:10.3390/gels4020028). The diameter of the P(NIPAM-*co*-AA) encapsulated nanoshells decreased as the solution temperature was increased, indicating the collapse of the hydrogel layer with increasing temperatures. Further, the surface plasmon resonance peak of the hydrogel-coated nanoshells appeared at ~800 nm, which lies within the tissue-transparent range that is important for biomedical applications. Including a silica core into thermo-responsive PNIPAAm microgels enhances their mechanical robustness. Birgit Fischer et al. showed that by varying the concentration gradient of the crosslinker, the thermo-responsive behavior of the core-shell microgels can be tuned with three different temperature scenarios (doi:10.3390/gels3030034). The further synthesis of stimuli-responsive colloidal nanocomposite hydrogels is reported by Avinash J. Patil et al., achieved by exploiting non-covalent interactions between anionic cellulose nanocrystals and polycationic delaminated sheets of aminopropyl-functionalized magnesium phyllosilicate clays (doi:10.3390/gels3010011). The preparation of hydrogel nanocomposites containing silver nanoparticles with a size of 15–21 nm is presented by G. Roshan Deen et al., achieved by diffusion and in-situ chemical reduction in chemically crosslinked polymers based on *N*-acryloyl-*N*′-ethyl piperazine (AcrNEP) and NIPAM (doi:10.3390/gels1010117). The polymer chains of the hydrogel network enabled the control and stabilization of silver nanoparticles without the need for additional stabilizers. The silver nanoparticles present in the nanocomposite offered additional physical crosslinking, which influenced media diffusion and penetration velocity. Finally, the preparation of PNIPAM hydrogels containing carboxymethylcellulose (CMC) and CMC/Fe_3_O_4_ nanoparticles is reported by Andrea Atrei et al. (doi:10.3390/gels2040030). FESEM images show that the CMC in the PNIPAM hydrogels induces the formation of a honeycomb structure. This surface morphology was not observed for pure PNIPAM hydrogels prepared under similar conditions. Both PNIPAM/Fe_3_O_4_ and PNIPAM/CMC/Fe_3_O_4_ hydrogels exhibit a superparamagnetic behavior.

